# Spectral Characteristic, Storage Stability and Antioxidant Properties of Anthocyanin Extracts from Flowers of Butterfly Pea (*Clitoria ternatea* L.)

**DOI:** 10.3390/molecules26227000

**Published:** 2021-11-19

**Authors:** Xueying Fu, Qiang Wu, Jian Wang, Yanli Chen, Guopeng Zhu, Zhixin Zhu

**Affiliations:** 1 Key Laboratory for Quality Regulation of Tropical Horticultural Crops of Hainan Province, College of Horticulture, Hainan University, Haikou 570228, China; fuemily@126.com (X.F.); wuqiangccb@163.com (Q.W.); chen_rose_79@163.com (Y.C.); guopengzhu@163.com (G.Z.); 2 Key Laboratory of Germplasm Resources Biology of Tropical Special Ornamental Plants of Hainan Province, College of Forestry, Hainan University, Haikou 570228, China; wjhainu@hainanu.edu.cn

**Keywords:** *Clitoria ternatea*, blue anthocyanins, ternatins, absorption spectrum, quinonoid base, stability, temperature, pH, antioxidant

## Abstract

Anthocyanins from flowers of the butterfly pea (*Clitoria ternatea* L.) are promising edible blue food colorants. Food processing often faces extreme pHs and temperatures, which greatly affects the color and nutritional values of anthocyanins. This study explored the color, spectra, storage stability, and antioxidant properties of *C. ternatea* anthocyanin extract (CTAE) at different pHs. The color and absorption spectra of CTAEs at a pH of 0.5–13 were shown, with their underlying structures analyzed. Then, the storage stability of CTAEs were explored under a combination of pHs and temperatures. The stability of CTAE declines with the increase in temperature, and it can be stored stably for months at 4 °C. CTAEs also bear much resistance to acidic and alkaline conditions but exhibit higher thermal stability at pH 7 (blue) than at pH 0.5 (magenta) or pH 10 (blue-green), which is a great advantage in food making. Antioxidant abilities for flower extracts from the butterfly pea were high at pH 4–7, as assessed by DPPH free radical scavenging assays, and decreased sharply when the pH value exceeded 7. The above results provide a theoretical basis for the application of butterfly pea flowers and imply their great prospect in the food industry.

## 1. Introduction

Due to the health concerns associated with synthetic pigments, the food industry has begun to invest heavily in natural food colorants [1,2]. Common plant pigments, including anthocyanins, betalains, and carotenoids, possess a red hue (from yellow to purple) and have been recommended for use as healthy natural food colorants [3,4]. However, among the above plant pigments, only a small fraction of anthocyanins are blue in color [2,3]. At present, the blue color in food is mainly presented by Brilliant blue FCF (derived from petrochemicals), and natural edible blue food colorants are very rare [1,2]. Problems regarding anthocyanin stability hinder its application as a food colorant [5,6].

Anthocyanins are a group of bio-active water-soluble flavonoids widely distributed in the plant kingdom that can render plant tissues with hues of pink, red, purple, and blue [3,7]. They are also beneficial to human health, possessing antioxidant and anti-inflammatory properties, as well as the ability to prevent tumors, diabetes, and many other cardiovascular diseases [4,6,8,9]. Anthocyanins, which are safe and highly nutritional, have great value and potential in food coloring. However, due to the unstable link between O1 and C2 at the C ring, anthocyanins in nature are extremely unstable and susceptible to degradation by external factors such as temperature, pH, light, oxygen, enzymes, metal ions, etc [2,5,6]. Food processing often faces extreme pHs and temperatures, and degradation and discoloration tend to occur during storage and processing of anthocyanins [10,11,12]. All the above stability-related problems bring great obstacles for the preservation of anthocyanins.

Blue anthocyanins are not rare in nature, as indicated by the variety of blue flowers [3,13]. However, the blue shades of anthocyanins mainly appear in the pH range of 5–7 by the quinonoid base form, which would easily degrade [2,14]. The stability problem for blue anthocyanins is even more serious for red hue anthocyanins. Luckily, there are always exceptions in nature. Butterfly pea (*Clitoria ternatea* L.), with blue flowers similar to butterflies, are often used for making blue cake or drinks in Southeast Asian countries such as Malaysia and the Philippines [13,15]. Ternatins, the special polyacylated anthocyanins from *C. ternatea*, are responsible for the blue hue [13,16,17,18]. The structure of ternatins is characterized as malonylated delphinidin 3,3′,5′-triglucosides, having 3′,5′-side chains with alternating D-glucose and *p*-coumaric acid units [16,17,18]. The high degree of glycosylation and acylation greatly improve the stability of ternatins [6,13]. In addition to the health benefits of anthocyanins, ternatins are of strong stability and have important potential as natural functional dyes for food or cosmetic products [19,20].

The special properties of *C. ternatea* anthocyanins have drawn researchers’ attention, and their stability at various pHs and temperatures has been studied [6,15,21,22]. However, the specific structures of ternatins at different pHs have not been clearly described, and most of the reported studies of storage stability are within a time range of one month. The combined influence of pH and temperature on the stability of anthocyanins has not been fully studied. It is also necessary to elucidate the effect of pH on the antioxidant activity for flowers of *C. ternatea*. Here, the color, spectra, storage stability, and antioxidant properties of *C. ternatea* anthocyanin extract (CTAE) were explored. Stability of CTAE was compared with four other common fruits or vegetables. The color and absorption spectra of CTAEs at different pHs were shown, with their underlying structures analyzed. Then the stability of CTAEs were explored under combinations of pHs and temperatures. Finally, the antioxidant capacity of *C. ternatea* flower extracts at different pHs were assessed by DPPH (2,2′-diphenyl-1-picrylhydrazyl) free radical scavenging abilities. From our results, CTAEs showed bright blue color and had the highest stability and antioxidant properties under neutral conditions. Anthocyanins from the flowers of the butterfly pea have great practical prospects in the food industry.

## 2. Results and Discussion

### 2.1. Stability Comparison of Anthocyanin Extracts from Five Plants

**Figure 1:**
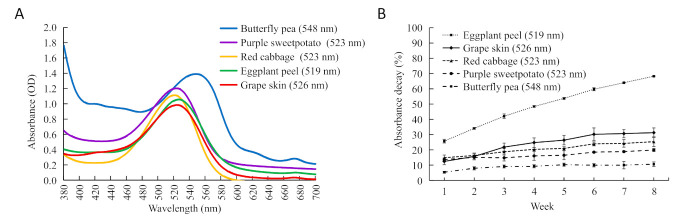
Absorption spectrum scanning (**A**) and the percent of absorbance decay (**B**) for the anthocyanin extracts from blue flowers of butterfly pea and four other common fruits and vegetables. The wavelength value in the bracket indicates the λmax in (**A**) and the measurement wavelength in (**B**) for each plant material.

Comparison of anthocyanin stability were conducted for *C. ternatea* and four other common vegetables or fruits, i.e., purple sweetpotato, red cabbage, grape skin, and eggplant peel. From the spectral scanning of their anthocyanin extracts, the absorption peak (λmax) of *C. ternatea* flowers was at 548 nm within the 400–680 nm visible region, deviating by about 23 nm from the λmax around 525 nm for the other plants (Figure 1A). This deviation in absorption peaks indicates structure variations, in accordance with the report that polyacylation can cause bathochromic shift for anthocyanins [23].

In dark conditions at room temperature (23–27 °C), the percent of absorbance decay at λmax were measured (Figure 1B). The anthocyanin stability of the five materials ranked as butterfly pea flowers > purple sweetpotato > red cabbage > grape skin > eggplant peel. At the 8th week, the percent of absorbance decay for *C. ternatea* flowers was the lowest (10.61%) and there was no obvious color change, while obvious color decay was observed for the other four plant materials. The eggplant peel extracts had the greatest degree of color fading, and its absorbance decay percent reached 68.22% at the 8th week.

### 2.2. Color and Spectral Characteristic of Anthocyanin Extract from C. ternatea Blue Flowers

**Figure 2:**
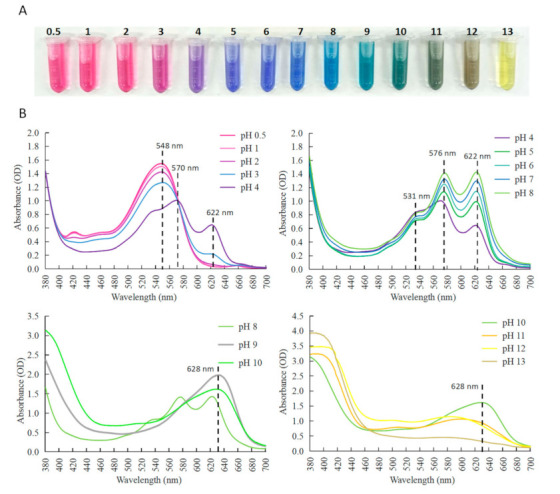
Color and absorption spectra of CTAEs at different pHs. (**A**) Color of CTAEs from pH 0.5 to 13. (**B**) Absorption spectra of CTAEs from pH 0.5 to 13. The absorption peaks are highlighted by vertical lines with values of λmax presented.

With the change of pH, anthocyanins will undergo reversible structural transformation, leading to color variations [14]. The CTAEs underwent gradual color change from magenta to purple, blue, green, and yellow from pH 0.5 to 13 (Figure 2A), which is of high accordance with the colors presented by Faezah et al. [22]. The corresponding absorption spectra were shown (Figure 2B).

From pH 0.5 to 3, the CTAEs were magenta (λmax at 548 nm), and the peak value decreased under higher pHs. From pH 3 to 4, the color changed from magenta to purple, and the absorption peak showed clear bathochromic shift and split into two peaks at 570 nm and 622 nm, with a shoulder peak at 531 nm. From pH 5 to 8, the CTAEs were bright blue, and the peaks at 576 nm and 622 nm lifted slightly as the pH increased. At pH 9, another bathochromic shift occurred, and the three peaks merged into one single peak around 628 nm. Then from pH 9 to 13, the peak around 628 nm declined. Notably, the absorbance within 380–400 nm was basically unchanged from pH 0.5 to 8, but elevated drastically from pH 8 to 13, indicating that yellow chalcone forms when pH > 8.

For the substance to produce a true blue color, the absorbed wavelength of light should be relatively long around 580–620 nm, which requires large and complex conjugated compounds [2]. From the absorption spectra, the CTAEs at pH 4–8 had high absorption within 580–620 nm, with corresponding liquid color as bright blue. At pH 9–10, the peak at 576 nm disappeared, and the peak at 628 nm and the absorbance around 380–440 nm gave the solution a blue-greenish color (green can be derived from yellow plus blue). The pH dependent structural transformation may result in color changes during storage [24]. Here, it can be concluded that CTAEs appear blue-green at the wide range of pH 4–10, which is an advantage of anti-discoloration in food making.

### 2.3. Speculated Structural Transformation of Ternatins with pH Variations

**Figure 3:**
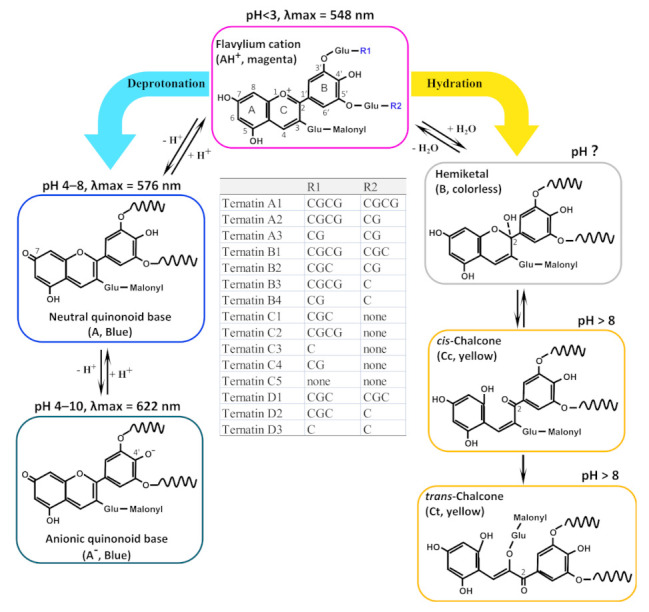
Speculated structural transformation of ternatins with pH variations. In the structure diagrams, D-glucose is simplified as Glu. The inset table shows the side chain variations of the 15 ternatins (A1–A3, B1–B4, C1–C5, and D1–D3) according to Terahara et al. [16,17,18], in which G is D-glucose and C is *p*-coumaric acid. The 3′ and 5′ side chains are simplified as wavy lines in anthocyanin forms other than AH^+^. The blue and yellow arrows show the two transformation directions for AH^+^, i.e., deprotonation and hydration, respectively. Speculated pH range for each anthocyanin forms were shown. However, as CTAEs never displayed colorless, a question mark was used for the unclear pH range for the colorless hemiketal form.

Anthocyanins in the solution exist as a multistate equilibrium, which is the structural basis of color change as the pH varies. The red hue flavylium cation (AH^+^) is favored at very acidic pH values (about pH < 2 for usual anthocyanins) [2,14]. Based on the color and the spectra of CTAEs (Figure 2), speculated structural transformation of ternatins as a function of pH were shown (Figure 3). It can be inferred easily that ternatins mainly adopt the magenta AH^+^ form (λmax = 548 nm) at pH < 3. When the pH increases, there are two transformation directions for the AH^+^, i.e., deprotonation and hydration. (**1**) When deprotonation occurs, C7-OH and C4′-OH will lose hydrogen sequentially to form the blue quinonoid base forms (A and A^−^), accompanied by two shifts in λmax––the first (AH^+^→A) typically of 20–30 nm and the second (A→A^−^) a further shift of 50–60 nm [14]. Here, spectra of ternatins correspond almost perfectly to the above patterns: 576 nm (by A) is a shift of 28 nm from 548 nm (by AH^+^), and 622 nm (by A^−^) is a shift of 46 nm from 576 nm (by A). The derived quinonoid base (A and A^−^) are blue or green (happens around pH 6–8 for usual anthocyanins) [2,14]. From our data, ternatins are speculated to take the forms of A and A^−^ at pH 4–8. The proportion of A decreased drastically at pH 9–10. (**2**) Hydration occurs at the position of C2 (typically at pH 4–5 for usual anthocyanins), leading to the colorless form of hemiketal (B). The B form will further isomerize into yellow chalcones when pH > 8 [2,14]. Here, from the absorbance increase within 380–400 nm from pH 8 to 13 (Figure 2), we can infer that the yellow chalcones also occurred at pH > 8 for ternatins. It should be noted that there are *cis*-chalcones (Cc) and trans-chalcones (Ct), and the transition from Cc to Ct is irreversible, which caused the eventual isomerisation of anthocyanins into yellow solutions. Additionally, as CTAEs never appeared colorless, the unclear pH range for the colorless hemiketal form was shown with a question mark (Figure 3).

To stabilize the blue quinonoid base forms (A and A^−^), anthocyanins in blue flowers or fruits often adopt strategies such as intra- or inter- molecular copigmentation, metal chelation, etc [2,23]. In *C. ternatea*, ternatins have long 3′ and 5′ side chains (shown by the inset table of Figure 3) [16,17,18]. Acylation on anthocyanin molecules can increase stability through intra- and/or inter-molecular copigmentation, and self-association reactions, by blocking hydration at C2 [2,23]. It can easily be inferred that ternatins adopt intra-molecular stacking to a very large extent, a strategy less affected by an ambient environment and much more reliable than inter-molecular stacking or metal chelation. This is the most possible reason that ternatins render a blue color within the wide range of pH 4–10 with strong stability.

### 2.4. Combined Effects of pH and Temperature on the Storage Stability of CTAEs

**Figure 4:**
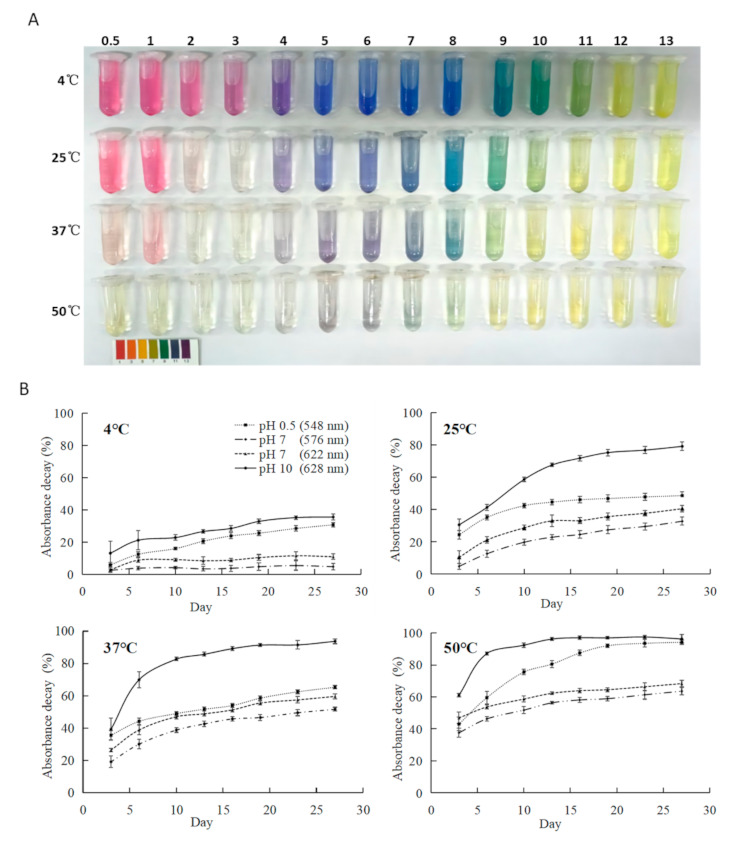
Combined effects of pH and temperature on the stability of CTAEs. (**A**) Color of CTAEs after 65 days’ storage in dark at 4, 25, 37, and 50 °C, respectively. Below is the color reference by universal pH indicator paper. (**B**) Absorbance decay percentages of CTAEs at pH 0.5 (λmax at 548 nm), pH 7 (λmax at 576 nm and 622 nm) and pH 10 (λmax at 628 nm) during 27 days’ storage in dark at 4, 25, 37, and 50 °C.

Foods often undergo heat or pH treatment during processing, which will greatly affect the color and content of anthocyanins [5,10,11,12]. To assess the combined effects of pH and temperature on the stability of CTAEs, four sets of temperatures (4 °C, 25 °C, 37 °C, and 50 °C) were applied to a series of CTAEs at a pH of 0.5–13 (Figure 4). The color of CTAEs after 65 days’ storage under combinations of pH and temperature were shown (Figure 4A). It can be clearly drawn that CTAEs are more stable under lower temperatures. CTAEs stored at 4 °C maintained a bright color and the color decay was not easy to distinguish with the naked eye when pH < 8, while for those stored at 50 °C, only CTAEs at pH 4–8 retained some blue-green hue. For CTAEs at pH 9–13, the color turned to bright yellow, indicating yellow chalcones (Cc and Ct). The production of Ct causes irreversible yellowing of the solutions. For CTAEs with pH < 3, the yellow hue was much paler, indicating partial degradation of anthocyanins into smaller colorless molecules, possibly due to the direct breaking up of anthocyanins into smaller colorless molecules. It is a little surprising that CTAEs were most stable under pH 4–8, when usual anthocyanins from other plants will continuously undergo irreversible formation of yellow Ct chalcone by the multistate equilibrium [2]. The above result must be realized by the long 3′ and 5′- polyacylated side chains of the ternatins. The pH 4–8 around neutral condition is also the pH range for most of our consumed food, which indicate great practical usage of *C. ternatea* in food industry.

Quantitative color decay were measured by OD values at their absorption peaks for representative pH solutions at 0.5, 7, and 10 (Figure 4B). OD_548_ (by AH^+^) were measured for CTAEs at pH 0.5. OD_628_ (mainly by A^−^) were collected for CTAEs at pH 10, while for CTAEs at pH 7, OD_576_ (by A) and OD_622_ (by A^−^) were both measured. It was clearly demonstrated that the increase in temperature accelerated the degradation of anthocyanins, also shown by Figure 4A, while under the same temperature, the peak stability of CTAEs ranked as pH 7 (576 nm) > pH 7 (622 nm) > pH 0.5 (548 nm) > pH 10 (628 nm). On the 27th day, the percentages of absorbance decay for peaks of pH 7 (576nm) were 4.7%, 32.8%, 51.7% and 63.6% at 4 °C, 25 °C, 37 °C, and 50 °C, respectively. In conclusion, the quinonoid base (A and A^−^) form of ternatins was very stable under a pH of around 7, possibly owing to the protection by the long 3’ and 5’- polyacylation side chains. The butterfly pea anthocyanin extract can be most stably stored under low temperatures and neutral pH conditions, while alkaline conditions (pH > 8) should be avoided.

### 2.5. Kinetic Parameters of Thermal Degradation for CTAEs at Different pHs

The stability of anthocyanins can be better understood by parameters of thermodynamic degradation kinetics. Thermal degradation kinetics were analyzed for the CTAEs (pH 0.5, 7, and 10) at different temperatures (4, 25, 37, and 50 °C), which showed degradation following the first-order reaction kinetics (Figure S1). The value of k (the 1st order rate constant) and t_1/2_ (time of half-life) were calculated (Table 1). Higher values of k means faster degradation of anthocyanins, while a higher value of t_1/2_ reflects better stability of the anthocyanins. Here, when the pH is fixed, the k value is increased, and the t_1/2_ is declined gradually with the elevation of temperature from 4 °C to 50 °C. The t_1/2_ values of the CTAEs (pH 0.5, 548 nm) at 4, 25, 37, and 50 °C were 50.9, 28.1, 17.7, and 5.6 days respectively, while the t_1/2_ values of the CTAEs (pH 7, 576 nm) were 334.2, 47.1, 26.0, and 18.5 days respectively. This result showed faster degradation of CTAE under higher temperature, and CTAE can be stored stably for months at 4 °C.

For CTAEs stored under the same temperature, k values ranked as pH 7 (576 nm) < pH 7 (622 nm) < pH 0.5 (548 nm) < pH 10 (628 nm). A lower k value corresponds to a higher value of t_1/2_, which reflects better stability of the CTAE. From these thermodynamic parameters, the stability of different forms of ternatins were indicated to rank as the quinonoid base form A (pH 7, 576 nm) > the quinonoid base form A^−^ (pH 7, 622 nm) > AH^+^ (pH 0.5, 548 nm) > A^−^ at pH 10 (pH 10, 628 nm). The t_1/2_ value for the form A (576 nm) at pH 7 (334.2 days) is much longer than that observed for the form AH^+^ (548 nm) at pH 0.5 (50.9 days). It is widely believed that anthocyanins are most stable at very low pH [2]. Here, the result that CTAEs were more stable at pH 7 may be related to the fact that intra-molecular stacking is mainly adopted by ternatins around a neutral pH range.

### 2.6. Antioxidant Properties for Extracts from C. ternatea Flowers

**Figure 5:**
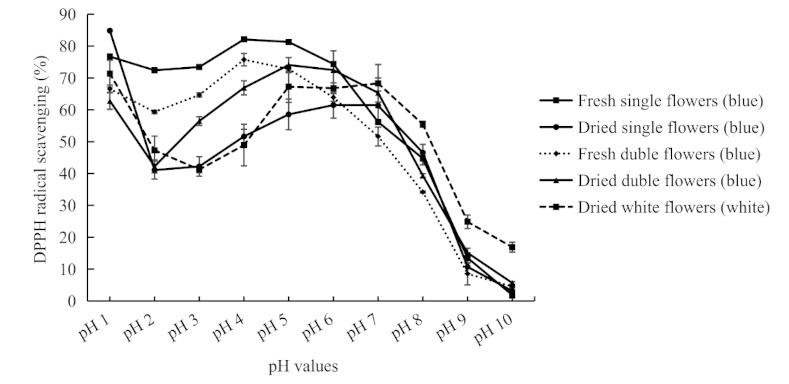
DPPH radical scavenging percentage for extracts of *C. ternatea* flowers at different pHs.

Oxidation is critical to the biological processes of energy production. However, uncontrolled production of free oxygen radicals is harmful to cells and would promote disease and aging [8,9,25]. Flavonoids, including anthocyanins, can scavenge free radicals and prevent oxidative stress and inflammation [26,27,28]. Here, DPPH free radical scavenging abilities were used to assess the antioxidant capacity of *C. ternatea* flower extracts at different pHs (Figure 5). Five kinds of flowers were used, including the fresh or dried blue single flowers, the fresh or dried blue double flowers, and the dried white flowers. The trends of scavenging percentages were similar for the five materials–high at pH 1, then decreased somewhat at pH 2–3, and then reached a smooth peak around pH 4–7. When the pH values were higher than 7, the scavenging percentages declined sharply with the elevation of pH values. When at pH 1 and pH 4–7, the percentage of radical scavenging can be up to over 80%. While at pH 2, the scavenging percentages were within the range of 41–72%. For extracts at pH 10, the scavenging percentages were <6% for blue flowers and were 17% for dried white flowers. Simply put, antioxidant properties for flower extracts from the butterfly pea were high at pH 4–7 and then decreased sharply when the pH exceeded 7. The above results may be related to the structural change of ternatins under various pHs. Studies on antioxidant activity of anthocyanin extract from litchi pericarp showed that anthocyanin extract had low antioxidant activity at pH 7 [29]. This inconstancy may reflect the general situation for usual anthocyanins, which were very unstable under neutral condition.

As for the five materials, the antioxidant capacity of fresh flowers was stronger than that of dry flowers when pH ≤ 4, while dry flowers showed slightly higher antioxidant capacity under neutral conditions. However, newly collected flowers were hard to preserve fresh for more than a few days. It can be drawn that although flowers freshly picked from plants have good antioxidant activity, dried flowers are more suitable for commercial usage.

Notably, white flowers also showed strong scavenging ability, probably because of the high content of other colorless flavonoids it possesses. Studies on lines of *C. ternatea* with different colors have shown that the content of myricetin glycosides (belonging to flavonols) in white flowers was 30–70 times of those in blue flowers [13], which supports our assumption. However, white flowers lack anthocyanins and do not have much use as food colorants.

## 3. Materials and Methods

### 3.1. Materials

For a stability comparison of anthocyanin extracts among various plants, purple sweetpotato, red cabbages, grapes, and eggplants were bought from the local market near the campus of Hainan University.

For *C. ternatea*, whole flowers including sepals were used, just as sold in the market in Southeast Asia. Five kinds of *C. ternatea* flowers were used, including the fresh or dried blue single flowers, the fresh or dried blue double flowers, and the dried white flowers. The fresh blue flowers were collected on the campus of Hainan University and dried in an oven at 40 °C for 24 h to achieve dried blue flowers. The dried white flowers were purchased online from the region of Negeri Selangor in Malaysia, which were dried in the sun. The fresh single blue flowers were the wild type and most widely distributed [13], and used for exploration of the color, spectra and stability of CTAEs. To provide theoretical reference for the application of *C. ternatea* flowers, all five kinds of flowers were assayed in the antioxidant evaluation to compare the antioxidant properties among different flower forms.

### 3.2. Stability Comparison for Anthocyanin Extracts from Five Plants

Anthocyanins were extracted from five plant materials, including fresh blue single flowers of *C. ternatea*, tubers of purple sweetpotato, leaves of red cabbage, skin of grape, and peel of eggplant. About 1–5 g plant materials were ground with 20 mL 1% HCl-methanol, and then filtered. As the usual anthocyanins would render an absorption peak of around 520 nm, the supernatants were collected, and measured for values of OD_520_ with 1% HCl-methanol as blank (UV-2100, UNICO). Appropriate dilutions were applied to the supernatants so that the final OD_520_ reached values around 1. The derived samples were subjected to continuous spectral scanning from 380–700 nm (UV-2600, SHIMDZU), and λmax within the 400–680 nm visible region was determined for extracts from each plant material.

The anthocyanin extracts were then placed in the dark at room temperature (23–27 °C) and measured for absorbance at λmax every week until the 8th week.

Absorbance decay percent (%) = [(A_0_ − A_t_)/A_0_] × 100%, (1)

A_0_ is the initial absorbance at λmax; A_t_ is the absorbance at λmax at the time of t. The data of three biological replicates were collected. The means and standard error (SE) of the three replicates were presented.

### 3.3. Stability Analysis for Anthocyanin Extracts from C. ternatea

About 40 g flowers of *C. ternatea* were extracted with 400 mL 1% HCl-methanol, and the supernatants were collected by filtering. OD_548_ were measured with 1% HCl-methanol as blank. Appropriate dilutions were applied to the supernatants so that the final OD_548_ reached values around 1. The initial pH of the derived extracts was about 0.5, and 0.1 mol/L or 1 mol/L NaOH was used to titrate the solutions to higher pHs. While titrating, the solution was shaken slightly and monitored for pH change.

For observation of color decay during storage, four aliquots of 2 mL solution were taken into four separate fresh tubes when the pH reached an integer from 1 to 13. The tube series of different pHs were arranged in order to take pictures, and then subjected to spectral scanning from 380–700 nm. Then the four tube series were placed in the dark at 4 °C, 25 °C, 37 °C, and 50 °C to further survey the color decay. This assay was repeated three times to ensure the color change.

For quantitative measurement of color decay, CTAEs of representative pHs at 0.5, 7, and 10 were chosen to be measured at their absorption peaks. The experimental process was the same as for the color observation above. While titrating, four aliquots of 10 mL solution were taken into fresh tubes when the pH reached 0.5, 7, and 10. Then the tubes were placed in dark at 4 °C, 25 °C, 37 °C, and 50 °C. For every three or four days, the OD values were measured at their absorption peaks pH 0.5 (OD_548_), pH 7 (OD_576_ and OD_622_), and pH 10 (OD_628_). Data of three independent repeats were collected until the 27th day. Absorbance decay percentages were calculated using the formula shown above. The means and SE of the three replicates were presented.

### 3.4. Thermal Degradation Kinetics

The thermal degradation kinetics were analyzed for the CTAEs (pH 0.5, 7, and 10) at different temperatures (4, 25, 37, and 50 °C) according to the method by Kirca et al. [30]. The rate constant of CTAE degradation (k) and half-life time (t_1/2_) were calculated with Equations (2)–(3).

ln(A_t_/A_0_) = −k × t, (2)

t_1/2_ = −ln0.5 × k^−1^, (3)

In the equations, A_t_ is the absorbance at time t (day); A_0_ is the initial absorbance.

### 3.5. Measurement of Antioxidant Activities for Extracts of C. ternatea at Different pHs

*C. ternatea* flowers of five different forms were assayed for antioxidant activities by methods of DPPH (2,2′-diphenyl-1-picrylhydrazyl) radical scavenging. The dried blue flowers were derived from collected fresh flowers. About 0.1 g of dry flowers can be derived from 1 g fresh of flowers.

One gram of fresh flowers or 0.1 g of dried flowers were extracted with 50 mL 75% ethanol, and the supernatants were collected by filtering. The initial pH of the extracts varied from 6.67 to 7.42. Half of the extracts were titrated by NaOH to higher pHs, and the other half were titrated by HCl to lower pHs. 2mL aliquots were taken to fresh tubes for solutions at pH 1–10 for further usage.

DPPH radical scavenging abilities were determined as Liu et al. [25]. Briefly, DPPH working solutions (1 × 10^−4^ mol/L) were prepared on the day of the assay, by dissolving 3.9432 mg DPPH in 100 mL absolute ethanol. Then, 500 μL sample were mixed with 1950 μL DPPH working solution (sample:DPPH =1:3.9) and 75% ethanol was used as the control sample. The mixed solutions were placed in dark at room temperature. OD_517_ were measured after 30 min.

Percentage of DPPH radical scavenging (%) = [(A_blank_ − A_sample_)/A_blank_] × 100%, (4)

A_blank_ represents the absorption of the control sample, A_sample_ represents values for tested samples. The data of three independent replicates were collected. The means and SE of the three replicates were presented.

## 4. Conclusions

Compared with many other plants, anthocyanins from flowers of the butterfly pea have very strong stability. CTAE render the color of blue-green within a wide range of pH 4–10. The stability of CTAE declined with the increase in temperature, and it can be stored stably for months at 4 °C. Surprisingly, in terms of pH, CTAEs were most stable under pH 4–8, and exhibited higher thermal stability at pH 7 (blue) than at pH 0.5 (magenta) or pH 10 (blue-green). Tests of antioxidant properties for butterfly pea flowers also showed good scavenging ability for flower extracts around pH 4–7. In conclusion, anthocyanins from *C. ternatea* were very stable under low temperature and neutral mild pH conditions and possess good antioxidant activity at pH 4–7, the pH range for most of our consumed food. Blue flowers of *C. ternatea* are good candidates for natural blue colorants that can be added to food. In consideration of easier preservation, dried blue flowers are recommended for commercial use.

## Figures and Tables

**Table 1:** Kinetic parameters of thermal degradation for CTAEs at different combinations of temperatures and pHs.

Temperature (°C)	R^2^	k (d^−1^)	k (min^−1^)	t_1/2_ (d)
pH 0.5 (548 nm)				
4 °C	0.9684	1.36 × 10^−^^2^	9.45 × 10^−^^6^	50.9
25 °C	0.9132	2.47 × 10^−^^2^	17.14 × 10^−^^6^	28.1
37 °C	0.9801	3.93 × 10^−^^2^	27.27 × 10^−^^6^	17.7
50 °C	0.9949	12.46 × 10^−^^2^	86.54 × 10^−^^6^	5.6
pH 7 (576 nm)				
4 °C	0.9012	0.21 × 10^−^^2^	1.44 × 10^−^^6^	334.2
25 °C	0.9652	1.47 × 10^−^^2^	10.22 × 10^−^^6^	47.1
37 °C	0.9140	2.67 × 10^−^^2^	18.52 × 10^−^^6^	26.0
50 °C	0.9604	3.75 × 10^−^^2^	26.04 × 10^−^^6^	18.5
pH 7 (622 nm)				
4 °C	0.9072	0.67 × 10^−^^2^	4.66 × 10^−^^6^	103.4
25 °C	0.9209	1.93 × 10^−^^2^	13.37 × 10^−^^6^	36.0
37 °C	0.9121	3.37 × 10^−^^2^	23.38 × 10^−^^6^	20.6
50 °C	0.9608	4.27 × 10^−^^2^	29.65 × 10^−^^6^	16.2
pH 10 (628 nm)				
4 °C	0.9707	1.63 × 10^−2^	11.32 × 10^−^^6^	42.5
25 °C	0.9177	5.82 × 10^−^^2^	40.39 × 10^−^^6^	11.9
37 °C	0.9163	10.34 × 10^−^^2^	71.82 × 10^−^^6^	6.7
50 °C	0.9071	14.92 × 10^−^^2^	103.58 × 10^−^^6^	4.6

Values of k were provided both in d^−1^ and min^−1^ for easier comparison with data of other species.

## Data Availability

Not applicable.

## Supplementary Materials

The following are available online, Figure S1: Degradation kinetics of CTAEs at pH 0.5, 7 and 10 in dark at 4, 25, 37 and 50 °C.

## Sample Availability

Not available.
