# Supplementary material for: Spectral Characteristic, Storage Stability and Antioxidant Properties of Anthocyanin Extracts from Flowers of Butterfly Pea (Clitoria ternatea L.)
Source: Molecules. 2021 Nov 19;26(22):7000. doi: 10.3390/molecules26227000 (PMC8622631; doi:10.3390/molecules26227000)
Supplement: Supplementary file 1 [file molecules-26-07000-s001.zip › molecules-1453965-supplementary.pdf]

Figure S1

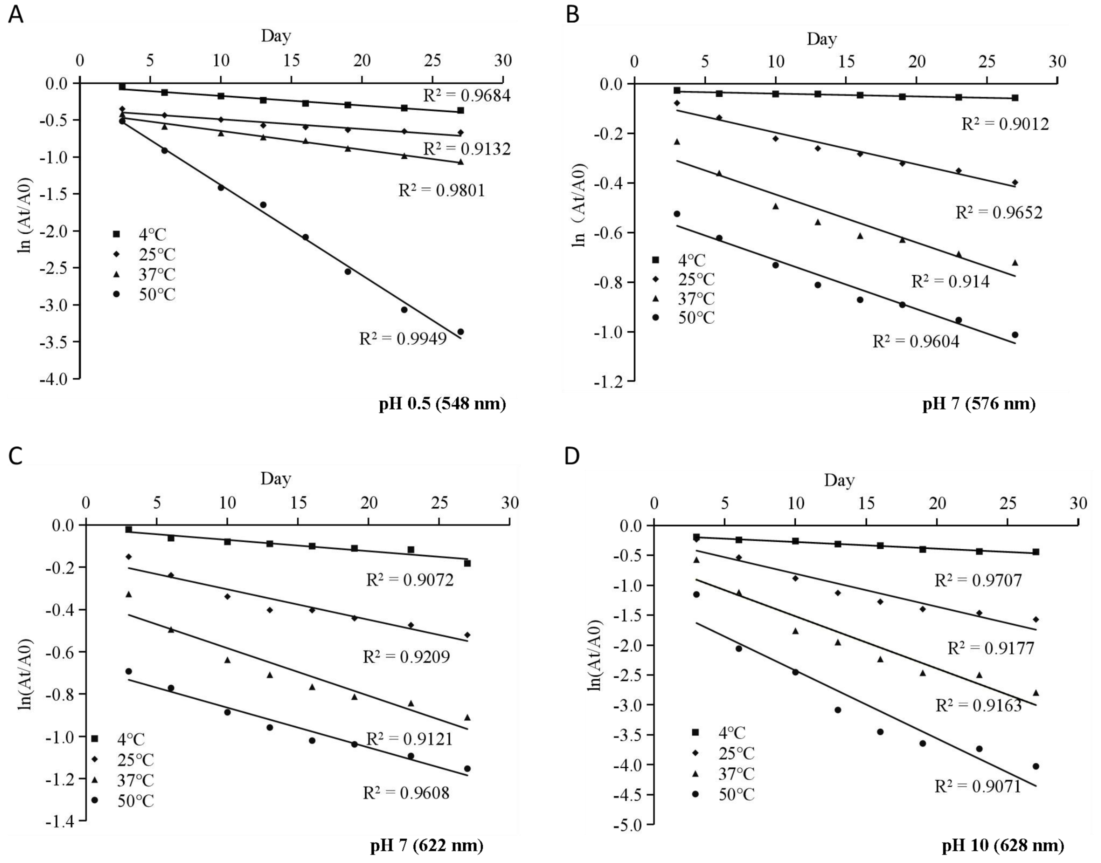

Figure S1. Degradation kinetics of CTAEs at pH 0.5, 7 and 10 in dark at 4, 25, 37 and 50°C. (A) Decay of OD_{548} for CTAEs at pH 0.5. (B-C) Decay of OD_{576} and OD_{622} for CTAEs at pH 7. (D) Decay of OD_{628} for CTAEs at pH 10.
